# Determinant and outcome of early diagnosis of HIV infection among HIV-exposed infants in southwest Ethiopia

**DOI:** 10.1186/1756-0500-7-309

**Published:** 2014-05-22

**Authors:** Gebremedhin Derebe, Sibhatu Biadgilign, Marina Trivelli, Gemechis Hundessa, Zinash D Robi, Mikael Gebre-Mariam, Misrak Makonnen

**Affiliations:** 1 Ethiopian Catholic Church, Health and HIV/AIDS Department, Addis Ababa, Ethiopia; 2 Independent Public Health Consultant, Addis Ababa, Ethiopia; 3 St. Luke Catholic Hospital and College of Nursing and Midwifey, Wolliso, Ethiopia; 4 IntraHealth International, Addis Ababa, Ethiopia

**Keywords:** Outcome, EID, HIV infection, HIV-exposed infants, Ethiopia

## Abstract

**Background:**

Preventing mother-to-child transmission (PMTCT) of human immunodeficiency virus infection (HIV) has been a fundamental advancement in the acquired immunodeficiency syndrome (AIDS) response for the past decade. Several countries have made great strides in the efforts to prevent HIV through mother-to-child transmission. The objective of this study is to assess the determinant and outcome of early diagnosis of HIV infection among HIV-exposed infants in southwest Ethiopia.

**Methods:**

An institutional based retrospective cohort study was conducted in a hospital. Medical records of HIV-exposed infants and their mothers enrolled into the program were reviewed. Data entry and analysis was carried out using SPSS version 20 for Windows.

**Results:**

A total of 426 HIV exposed infant-mother pairs where both mother and infants received a minimum ARV intervention for PMTCT were included in the study. Two hundred fifty-four (59.6%) of mothers had attended antenatal care (ANC). Of all participants, 234(54.9%) mothers did not receive any PMTCT prophylaxis during ANC, while only 104(24.4) received antiretroviral (ART) as PMTCT prophylaxis and 163(38.3%) claimed that did not observe any infant PMTCT interventions while 135(31.7%) of the infants received single-dose NVP + AZT. About 385(90.4%) infants were not infected at their final infection status. Those mothers who did not attended ANC follow-up, infants on mixed and complementary feeding and infants weaned off and mothers who were in WHO clinical stage III and IV were more likely to have HIV sero positive infant.

**Conclusion:**

This study showed that 385(90.4%) of the infants were not infected at their final infection status. Therefore, encouraging pregnant women to visit health facilities during their course of pregnancy, focusing on exclusive breast feeding counseling and promotion, and early initiation of antiretroviral treatment to HIV infected pregnant women are recommend.

## Background

In 2001, the United Nations General Assembly Special Session (UNGASS) placed a clear emphasis on the effect of HIV/AIDS on maternal and child health. The final declaration of commitment1from the assembly stated that the proportion of infants infected with HIV should be reduced by 20% by 2005, and by 50% by 2010. This goal was to be reached by ensuring that 80% of pregnant women who receive antenatal care have access to HIV-1 prevention services [1]. More than 90% of new paediatric HIV cases are acquired through mother-to-child-transmission [2]. Several studies have shown that child mortality increases substantially in children who are infected with HIV via their mothers [3-5].

Mother-to-child transmission (MTCT) of HIV accounts for 14% of all new HIV infections worldwide, and may occur during pregnancy, labor and delivery or breastfeeding. In the absence of prevention, rates of MTCT are estimated to be 25–35 percent [6,7]. Therefore, HIV counseling and testing for pregnant women is therefore considered as a key factor for the success of PMTCT [8,9]. Detection of maternal infection early in pregnancy through voluntary counseling and HIV testing (VCT) is critical for PMTCT [10]. PMTCT of HIV has been a fundamental advance in the AIDS response for the past decade. Infection rates among children born to mothers living with HIV, 370 000 children were infected with HIV in 2009. Seven other countries in sub-Saharan Africa have coverage levels of 50% to 80%. Sub-Saharan Africa as a whole achieved 54% [40%–84%] coverage [11].

Ethiopia has adopted the WHO/UNICEF/UNAIDS 4-pronged PMTCT strategy as a key entry point to HIV care for women, men and families [12]. According to Ethiopia Demographic and Health Survey (EDHS) 2011, the national adult HIV prevalence was 1.5% with women disproportionately infected (1.9% compared to 1.0% in men) [13]. The national estimate for HIV positive pregnant women needing PMTCT was 38,404 for the year 2012 [14]. In most resource-limited countries, PCR-based assays are not available for the early diagnosis of pediatric HIV. The high mortality rate of pediatric HIV in resource-limited countries is partly due to lack of early diagnosis and low coverage of pediatric highly active antiretroviral therapy (HAART) treatment. There is an urgent need to integrate low-cost and accessible viral nucleic acid based assays [15,16]. So the objective of this study is to assess the determinant and outcome of early diagnosis of HIV infection among HIV-exposed infants in southwest Ethiopia.

## Methods

### Study settings and context

Health institution-based retrospective cohort study was conducted at St. Luke Hospital from December 2009 to March 2010 to evaluate the determinant and outcome of EID (Early Infant Diagnosis) of HIV infection. The study was conducted in Woliso town, Southwest Shewa administrative zone; one of the eighteen zones in Oromia Regional State. Woliso is the capital city of the zone which is located 114 Km, South-west of Addis Ababa. The administrative zone has 11 rural districts. The hospital was established on January 1, 2001 [17].

### Study design and participants

Participants for this study were mother-child pairs registered from the start of the program of antiretroviral treatment service including PMTCT. Medical records of HIV-exposed infants and their mothers enrolled into the program were reviewed for study inclusion. To be eligible for inclusion, both mother and child had to be enrolled in the PMTCT program and their data available during data collection period. All HIV-positive women should be routinely assessed for ART eligibility and initiated on HAART if eligible, at the point of care or at HIV care/ART clinic ensuring the follow-up of comprehensive care and treatment for HIV exposed infants ranging from follow-up visits, counsel about infant feeding practices and support mother’s choice, provision of cotrimoxazole prophylaxis starting at 4–6 weeks old and DNA PCR testing at 6 weeks or as early as possible thereafter if not possible at 6 weeks [12].

### Data collection procedures and measurement

Information on demographic characteristics, HIV testing and counseling(HCT), and uptake of different components of the PMTCT package were obtained from HIV exposed infant follow-up cards, HIV care/ART follow up cards and intake forms, HCT, PMTCT registers and antenatal patient cards, abstracted demographic, clinical, and pregnancy outcome data from delivery and newborn records, using standard abstraction forms. Data extraction was made by the team of the investigators. Before data extraction process, all the investigators were all orientated on how to complete the structured data collection sheet. According to the revised guidelines of PMTCT, it is recommended that all women coming for ANC, labour and delivery and post partum follow-up, if not tested during current pregnancy should be routinely informed about the benefits of HIV testing for mother and baby in a group or individual basis and tested or offered testing. Care of newborn to HIV-positive Mothers states that follow up should be schedule at 6 hours, 6 days, 6 weeks, 10 weeks, 14 weeks, then monthly until 6 months, and thereafter every 3 months until 18 months if infant is asymptomatic and wherever possible carry out DNA PCR testing at 6 weeks or as early as possible thereafter if not possible at 6 weeks. If the child is less than 18 months of age, HIV virological test (DNA PCR using DBS) will be done to say DNA PCR test using DBS is done at 6 weeks to identify infected infants, not to exclude infection [12]. The electronic medical record of the mother and the corresponding infants were cross-checked for the availability of their data and were linked with infant records in order to confirm maternal prophylaxis data during the appointment period. To ensure quality of the data, the following measures were taken: pre testing of the questionnaire, checking of completed questionnaires before data entry, supervision of data entry and errors and consistency checking procedures for the data were controlled during analysis. The dependent (outcome) variable was the infant HIV status at the end of the program (18 months).

### Statistical analysis

The data extraction from the medical records was checked for completeness and consistency. Data entry and analysis was carried out using SPSS version 20 for Windows (SPSS, Chicago, IL, USA). Descriptive statistical methods, bivariate analysis and multivariable logistic regression analysis were used. Odds ratio with its corresponding 95% confidence interval was used as the measure of the degrees association with infant HIV positivity. P-value less than 0.05 were considered significant.

### Ethical considerations

Ethical clearance was obtained from the local Research and Publication Committee of the St. Luke Hospital (Ref.157/2012, issued 11 of June 2012). Since the study utilizes routinely collected, aggregated program data at the hospital, confidentiality of patient information was ensured as the names or identification number of study participants was not included in the data collection format.

## Results

A total of 426 mother-infant pairs where both mother and infants received a minimum ARV intervention for PMTCT. From this, 221(51.9%) of the infants were male gender. The detailed description of the demographic and clinical characteristics is described in Table 1.

**Table 1:** Demographic and clinical characteristics of the study participants, southwest Ethiopia, 2010

**Characteristics**	**Frequency(percentage)**
Gender	
Male	221(51.9)
Female	205(48.1)
Mothers attended ANC	
Yes	254(59.6)
No	114(26.8)
Not available	58(13.6)
Maternal CD4 count during ANC	
<200	46(10.8)
200-350	74(17.4)
>350	124(29.1)
Not done	2(0.5)
Not available	180(42.3)
Maternal clinical stage during ANC	
WHO stage I	171(40.1)
WHO stage II	46(10.8)
WHO stage III	27(6.3)
WHO stage IV	2(0.5)
Not available	180(42.3)
Type of PMTCT prophylaxis received by the mother during ANC	
Combined ARV prophylaxis	76(17.8)
ART	104(24.4)
Not documented	12(2.8)
None	234(54.9)
Mother’s place of delivery	
Home	137(32.2)
Health facility	250(58.7)
Not available	39(9.2)
Mode of delivery	
Caesarian section	10(2.3)
Spontaneous vaginal delivery	362(85.0)
Not documented	54(12.7)
Mothers took intra partum ARV prophylaxis	
Yes	247(58.0)
No	146(34.3)
Not documented	33(7.7)
Types of ARV received by mother during labor	
Single-dose NVP	68(16.0)
Combined ARV prophylaxis	77(18.1)
HAART	104(24.4)
None	145(34.0)
Not documented	32(7.5)
Type of infant PMTCT interventions	
Single-dose NVP	89(20.9)
Single-dose NVP + AZT	135(31.7)
Not documented	39(9.2)
None	163(38.3)
Infant feeding option chosen	
Exclusive replacement feeding(ERF)	23(5.4)
Exclusive breastfeeding (EBF)	342(80.3)
Not available	61(14.3)
Father’s HIV status	
Positive	188(44.1)
Negative	41(9.6)
Not tested	182(42.7)
Separated	15(3.5)
DNA PCR result of the infant	
Not done	65(15.3)
Negative	318(74.6)
Positive	31(7.3)
indeterminate pending	12(2.8)
Infant feeding practice	
Infant on exclusive breast feeding	99(23.2)
Infant is on exclusive replacement feeding	24(5.6)
Infant is on mixed feeding	22(5.2)
Infant is on complementary feeding	48(11.3)
Infant is weaned off	193(45.3)
Not documented	40(9.4)
Age at which complementary feeding is started	
Before 4 months	19(4.5)
4-6 months	88(20.7)
After 6 months	115(27.0)
Not documented	204(47.9)
Duration of breast feeding	
<6 months	9(2.1)
6-12 months	134(31.5)
12-18 months	41(9.6)
>18 months	11(2.6)
Not documented	231(54.2)
Maternal clinical stage during breast feeding	
WHO stage I	210(49.3)
WHO stage II	78(18.3)
WHO stage III	32(7.5)
WHO stage IV	6(1.4)
Not documented	100(23.5)
Maternal status during breast feeding	
On Pre ART	240(56.3)
On ART	102(23.9)
Not enrolled/Unknown	61(14.3)
Not applicable	23(5.4)
Maternal adherence to ART	
Good	92(21.6)
Fair	2(0.5)
Poor	3(0.7)
Mother on Pre ART	238(55.9)
Not applicable	91(21.4)
Maternal CD4 count during breastfeeding	
<200	35(8.2)
200-350	84(19.7)
>350	196(46.0)
Not documented	111(26.0)
Developmental pattern of the child	
Appropriate	343(80.5)
Delayed	40(9.4)
Not documented	43(10.1)
Growth pattern of the child	
Normal	315(73.9)
Abnormal	68(16.0)
Not documented	43(10.1)
Final infection status of the infant	
Infected	41(9.6)
Not infected	385(90.4)
Infant outcomes	
Discharged	171(40.1)
Lost	168(39.4)
Dead	6(1.4)
On HAART	26(6.1)
Still on follow up	47(11.0)
Transfer out	8(1.9)
Age at discharge from the programme	
<1 year	86(20.2)
12-18 months	73(17.1)
18 months	7(1.6)
>18 months	5(1.2)
Not documented	255(59.9)

Not available – There is no registered document in the patents file.

### ANC attendance and referral to care and treatment

Two hundred fifty-four (59.6%) of the mother had attended antenatal care (ANC). About 46(10.8%) of the mothers had <200 and 180(42.3%) unknown CD4 count during their ANC. Similarly, 171(40.1%) and 180(42.3%) were in WHO stage I and unknown maternal clinical stage during ANC respectively. From the participants, 234(54.9%) of them did not receive any PMTCT prophylaxis by the mother during ANC while only 104(24.4) receive ART as a PMTCT prophylaxis. About 250(58.7%) of mothers gave delivery in health facilities and while 137(32.2%) delivered at home. Majority of the participants 362(85.0%) delivered through spontaneous vaginal delivery. About 247(58.0%) of the mothers took intra partum ARV prophylaxis. One hundred four (24.4%) and 77(18.1%) of the mothers received HAART and combined ARV prophylaxis during labor respectively. Approximately 163(38.3%) of the participants claimed they did not attended any infant PMTCT interventions while 135(31.7%) of the infants received single-dose NVP + AZT. Interestingly, 343(80.5%) of the children were appropriate for their development for their age and 315(73.9%) of them had a normal growth pattern.

### Breastfeeding pattern

About 342(80.3%) of the mothers mentioned that Exclusive breastfeeding (EBF) is the chosen infant feeding option. Regarding the sero status of the father, 188(44.1%) and 182(42.7%) of the cases the result turn to be positive and not tasted respectively. About 318(74.6%) the cases, the result of DNA PCR result of the infant were negative. Concerning infant feeding practice, 193(45.3%) of the mother mentioned that their infant is weaned off where as 99(23.2%) as infant on exclusive breast feeding. About 115(27.0%) and 204(47.9%) of the infants started their complementary feeding at the age after 6 months and unknown duration. Significant proportion 88(20.7%) of infants were also started at about 4–6 months. From those who already started, 134(31.5%) them gave breast feeding for about 6–12 months duration. Nearly half of 210(49.3) of the mother were at WHO stage I during breast feeding. Similarly, 78(18.3%) of them were on WHO stage II. Likewise, 240(56.3) and 102(23.9) of the mothers were on Pre-ART and ART respectively during breast feeding. From those who started the ARV treatment, 92(21.6%) was good adherence status. About 196(46.0%) and 90(21.1%) of the participants have >350 and unknown CD4 count during breastfeeding.

### Outcome of the cohort

About 385(90.4%) of the infants were not infected at their final infection status. The final outcome of the children under this cohort were 171 (40.1%) discharged and 168(39.4%) lost. Similarly, the age at which the children were discharged from the program at 86(20.2%) and 73(17.1%) of the cases were <1 year and 12–18 month.

### Factor associated with prevalence of HIV infection among HIV-exposed infant

Those mother whose didn’t have ANC follow up were five times more likely to have HIV sero positivity infant than those mother who had ANC visits[ OR = 5.28, 95%CI =2.24, 12.45]. Those mother whose Infant is on mixed feeding were six times [OR = 6.63, 95% CI = 1.23, 35.56], Infant on complementary feeding ten times [OR = 10.04, 95% CI = 2.57, 39.22] and Infant weaned off were ten times more likely to have HIV sero positivity infant [OR = 10.43, 95% CI =2.10, 51.86] compared with Infant on exclusively breastfeeding. Mothers who were in WHO clinical stage III were six times [OR = 6.12, 95% CI =1.87, 20.07] and WHO clinical stage IV were ten times [OR = 10.23, 95%CI = 1.34, 78.0] more likely to have HIV sero positivity infant than those mothers who are WHO clinical stage I [Table 2].

**Table 2:** Multivariable logistic regression model for prevalence of HIV infection among HIV-exposed children in southwest Ethiopia, 2010

**Variables**	**Infection status**		**Crude OR (95% CI)**	**P-value**	**Adjusted OR (95% CI)**	**P-value**
	**Positive n (%)**	**Negative n (%)**				
Mother had ANC visits				0.001		0.001
Yes	11(4.3)	243(95.7)	1		1	
No	24(21.1)	90(78.9)	5.9(2.77, 12.5)		5.284(2.243, 12.447)	
Unknown	6(10.3)	52(89.7)	2.55(0.90,7.20)		2.373(0.576, 9.781)	
Infant feeding practice				0.001		0.011
Infant on exclusively breastfeeding	3(3)	96(97)	1		1	
Infant on replacement breastfeeding	2(8.3)	22(91.7)	2.91(0.46, 18.47)		3.452(0.458, 26.020)	
Infant on mixed breastfeeding	4(18.2)	18(81.8)	7.11(1.47, 34.5)		6.626(1.234, 35.561	
Infant on complementary breastfeeding	14(29.2)	34(70.8)	13.18(3.57,48.68)		10.039(2.569, 39.223)	
Infant weaned off	15(7.8)	178(92.2)	2.7(0.76,9.55)		10.427(2.097, 51.856)	
Unknown	3(7.5)	37(92.5)	2.59(0.50,13.44)		1.995(0.365, 10.898)	
Maternal clinical stage during breast feeding				0.004		0.014
Stage I	11(5.2)	199(94.8)	1		1	
Stage II	10(12.8)	68(87.2)	2.66(1.08,6.54)		1.633(0.587, 4.546)	
Stage III	8(25)	24(75)	6.03(2.21, 16.46)		6.123(1.868, 20.072)	
Stage IV	2(33.3)	4(66.7)	9.04(1.49,54.87)		10.226(1.341, 77.998)	
Unknown	10(10)	90(90)	2.01(0.82,4.90)		1.625(0.447, 5.902)	

## Discussion

The study demonstrated that 163(38.3%) of the participants claimed that didn’t observe any infant PMTCT interventions while 135(31.7%) of the infants received single-dose NVP + AZT. About 318(74.6%) the cases, the result of DNA PCR result of the infant were negative and 193(45.3%) of the mother mentioned that their infant is weaned off where as 99(23.2%) as infant on exclusive breast feeding. Those mothers who did not attended ANC follow-up, infants on mixed and complementary feeding and infants weaned off and mothers who were in WHO clinical stage III and IV were more likely to have HIV sero positive infant.

In most of the cases, without the intervention of any PMTCT program, 30-45% of infants born to HIV-positive mothers in developing countries become infected during pregnancy, delivery and breastfeeding [18]. In this study, two hundred fifty-four (59.6%) of the mother had attended antenatal care (ANC). This reflects the low usage of ANC service utilization hence only 234(54.9%) of them didn’t receive any PMTCT prophylaxis by the mother and those mother whose didn’t have ANC follow up were five times more likely to have HIV sero positivity infant than those mother who had ANC visits. Similar studies have shown that in Nigeria, seventy-three mother-baby pairs were not opportune to get ARV prophylaxis and 39 babies were PCR-positive (53.4%) [19] and patient attrition along with the lower ANC attendance rate of 58% observed in Nigeria, is likely to contribute to the suboptimal uptake of ARV prophylaxis for PMTCT [20]. In contrary to this, PMTCT ANC services are feasible in resource limited settings whereby 80% of ANC attendees accepted the HIV test and a majority of the HIV positive women commenced ARV prophylaxis [21,22]. So encouraging pregnant women to have ANC is a crucial step to eliminate the virtual HIV transmission to the newborn babies and achieving the HIV free generation from the globe.

In order to avoid the increased incidence of diarrhea, pneumonia, and death observed with replacement feeding, WHO recommends breastfeeding duration for 2 years or longer [23,24]. In this study, 99(23.2%) infant were on exclusive breast feeding practice. Breastfeeding in many settings may continue through the first 2–3 years of life, suggesting an important role for repeat testing to confirm HIV-negative diagnoses [25]. The probability of HIV positive infants were more pronounced in those who have started on complementary feeding and infant weaned off. In case, if infants are found to be uninfected, decisions to avoid early mixed feeding and to shorten the duration of breastfeeding to 12 months will suffice for prevention of HIV infections [26,27]. Similarly, the rate of mixed feeding is high and so the risk of MTCT is increased [28] and overall MTCT rate of 18.5% among breastfed babies, and 68.0% among mixed fed babies were found in Southeastern Nigeria [19]. Other study in south Africa also demonstrated that exclusive breastfeeding carried a lower risk of HIV transmission than mixed feeding and a similar risk to no breastfeeding in Durban, South Africa [29]. We found that 88(20.7%) of infants were also started complementary food about 4–6 months. In another study, in Rwandan national PMTCT programme, the majority of women in their study reported a HIV-free survival rate at nine to 24 months is similar to the 12-to 18-month HIV-free survival rate of 95% [30].

In Nigerian study, forty-seven babies (15.5%) were mixed-fed and thirty-two (68.0%) of them turned out PCR-positive. Two hundred and twenty-eight babies (75.0%) were never breastfed, out of which 11 (4.8%) were PCR-positive. Only 35.5% (27 out of 76) of babies were exclusively breastfed. Five (18.5%) of exclusively breastfed babies were found to be PCR-positive [19]. In another study, it is clearly demonstrated that in children > 6 months the positivity rate was significantly higher in breast fed 42.8% (3/7) as compared to non breast fed 5% (2/40) children [31]. The more likely explanation for this is that mixed breast feeding and early initiation of complementary feeding practice exacerbate the increasing vulnerability of HIV positivity to their infants. Our finding is also supported by most literatures in the area and the risks of mother-to-child transmission by breastfeeding duration which depends on studies with short breastfeeding durations, and difficult to distinguish exclusive from mixed breastfeeding [32].

Interestingly, in our study, we found that as the severity of maternal clinical stage is found to be associated with infants HIV positivity. Studies showed that children whose mothers had evidence of advanced HIV disease were at substantially increased risk of death [33] and 18-month HIV transmission [34] and coincide across morbidity, mortality, and growth of HIV-exposed, HIV uninfected children [35] and rapidity of disease progression [36,37], and transmit HIV [38,39]. This shows the fact that women might be recently infected or had more advanced disease and therefore transmitted higher levels of HIV to their infants and as well the virus strains in these women were more virulent and convey less effective passive immunity to their infants [40].

The finding of this study should be interpreted in light of some limitations. The recall bias on remembering of the breast feeding pattern, the retrospective nature of data collection might have some measurement error. We also anticipated for selection bias as the participants were actually found in the health institution whereby receiving some interventions. Despite this, the participants were followed over long period of time and the paucity of literature in the country particularly evaluating the effectiveness of PMTCT services, it shade a light on the quality and efficacy of the PMTCT.

## Conclusion

In conclusion, about 385(90.4%) of the infants were not infected at their final infection status. So encouraging pregnant women to visit health facilities during their course of pregnancy would contribute to increase the uptake of PMTCT interventional program, focusing on exclusive breast feeding counseling and promotion, and early initiation of antiretroviral treatment to HIV infected pregnant women contribute to the elimination of transmission to HIV to the new born babies and uninfected partners beyond the core asset of PMTCT services.

## Competing interest

There is no conflict of interest as a result of publication of this article.

## Authors’ contribution

GD conceived and designed the study, data extraction, performed analysis and interpretation of data and drafted the manuscript. SB, GH assisted the study design, data interpretation, drafted and critically reviewed the manuscript. MT, MG, ZD, MM assisted in interpretation of data, manuscript preparation and critically reviewed the manuscript. All authors read and approved the final manuscript.
